# Three-dimensional digital microfluidic manipulation of droplets in oil medium

**DOI:** 10.1038/srep10685

**Published:** 2015-06-02

**Authors:** Jiwoo Hong, Young Kwon Kim, Dong-Joon Won, Joonwon Kim, Sang Joon Lee

**Affiliations:** 1 Department of Mechanical Engineering, Pohang University of Science and Technology, San 31 Hyoja-dong, Nam-Gu, Pohang, Gyeongbuk, 790-784, South Korea

## Abstract

We here develop a three-dimensional DMF (3D DMF) platform with patterned electrodes submerged in an oil medium to provide fundamental solutions to the technical limitations of 2D DMF platforms and water–air systems. 3D droplet manipulation on patterned electrodes is demonstrated by programmably controlling electrical signals. We also demonstrate the formation of precipitates on the 3D DMF platform through the reaction of different chemical samples. A droplet containing precipitates, hanging on the top electrode, can be manipulated without adhesion of precipitates to the solid surface. This method could be a good alternative strategy to alleviate the existing problems of 2D DMF systems such as cross-contamination and solute adsorption. In addition, we ascertain the feasibility of temperature-controlled chemical reaction on the 3D DMF platform by introducing a simple heating process. To demonstrate applicability of the 3D DMF system to 3D biological process, we examine the 3D manipulation of droplets containing mouse fibroblasts in the 3D DMF platform. Finally, we show detachment of droplets wrapped by a flexible thin film by adopting the electro-elasto-capillarity (EEC). The employment of the EEC may offer a strong potential in the development of 3D DMF platforms for drug encapsulation and actuation of microelectromechanical devices.

Digital microfluidics (DMF) has been used to manipulate discrete droplets on an electrode array by utilizing dielectrophoresis (DEP) and electrowetting (EW)^1^,^2^,^3^,^4^,^5^. Here, EW controls the apparent contact angle by applying electrical signals^6^,^7^. It has been accepted as a suitable tool for basic fluidic operations, such as formation, transport, splitting or coalescence, and mixing of droplets, without the need for any moving micromechanical parts or external pumping systems^1^,^8^. The superiority of EW-based DMF (DMF in this work) over conventional techniques also involves reduced volumes of test samples and reagents, fast analysis, high throughput, and easy automation^1^,^2^,^3^. Based on these advantages, DMF has been successfully employed to numerous biochemical applications, such as cell culture/assays^9^,^10^,^11^ and DNA/protein analysis^12^,^13^,^14^,^15^,^16^.

Currently, DMF is commonly performed on two-dimensional (2D) platforms. Although 2D DMF has been widely used for practical applications, it still has some technical problems, such as cross-contamination^17^,^18^, solute adsorption, and resultant degradation of sensitivity in biochemical analysis^19^,^20^. Accordingly, the extension of 2D to three-dimensional (3D) DMF may provide fundamental solutions to the aforementioned problems. In addition, such extension will further corroborate the optimal routing of droplets, high-throughput analysis, and integrated device with high density.

Recently, several 3D droplet manipulation methods by means of EW were developed^21^,^22^,^23^. Yang *et al.*^21^ employed EW actuations to transport droplets on 3D structures that have tilted and curved surfaces. Abdelgawad *et al.*^22^ also handled droplets on various 3D structures, such as inclined, vertical, twisted, and upside-down plates. Based on these studies, Fan *et al.*^23^ constructed a DMF interface with a droplet-on-a-wristband consisting of a long, curved, and closed plane. However, these 3D manipulation methods have technical limitations because of the direct contact of the moving droplets with the solid surfaces, even though the droplets can be manipulated on 3D structures.

Droplet lift-off methods by means of EW were proposed to minimize the direct contact of the droplets with solid surfaces^24^,^25^,^26^. Lee *et al.* reported that a droplet can jump on a superhydrophobic surface by EW actuations with square and sinusoidal pulses^24^,^25^. Similarly, Lapierre *et al.* applied a modulated AC signal to bounce a droplet on a superhydrophobic surface^26^. More recently, Lee *et al.* extended their previous studies on droplet detachment from superhydrophobic surfaces^24^,^25^,^26^ by employing EW actuations with square pulses to detach droplets from a hydrophobic surface^27^. Wang *et al.*^28^ and Chae *et al.*^29^ constructed simple 3D DMF platforms with hydrophobic surfaces by applying EW-based lift-off methods.

However, all the aforementioned droplet lift-off methods were performed in an air medium^24^,^25^,^26^,^27^,^28^,^29^. Thus, they have several weak points, such as the requirement for high driving voltage and the difficulty in droplet manipulation at a high temperature because of droplet evaporation. These technical limitations can be overcome by introducing an immiscible fluid system (e.g., an aqueous droplet in oil medium) because the oil medium reduces contact-angle hysteresis and thus lowers the driving voltage^1^,^30^,^31^. The ambient oil also prevents droplet evaporation, which allows the droplet to be manipulated at a high temperature^1^,^32^. It likewise inhibits surface contamination caused by the adsorption of biomolecules^33^,^34^. These circumstances spontaneously require the development of a new droplet detachment method in immiscible fluids using EW actuations. To serve this purpose, we previously demonstrated the jumping of droplets in immiscible fluids (i.e., water droplet in oil and vice versa) using DC and square pulse EW actuations^35^. The critical conditions for droplet detachment in immiscible fluids were found to be functions of physical parameters, including droplet volume, applied voltage, and viscosity of the ambient fluid. In addition, we demonstrated the detachment of droplets containing a mixture of human umbilical vein endothelial cells and collagen submerged in silicone oil to get closer to the realization of a 3D DMF platform. A 3D DMF platform with patterned electrodes submerged in oil was also preliminarily developed to resolve the technical limitations caused by the use of a needle electrode.

This study experimentally determines the critical physical conditions for the vertical transport of droplets on patterned electrodes in oil medium (Fig. 1), such as electrical signal and threshold voltage. Based on the results, 3D manipulation (i.e., vertical and horizontal transport, and mixing) of droplets is established by programmably controlling electrical signals. The applicability of the present 3D DMF platform to chemical syntheses is also tested by examining the transport, merging, mixing, and detachment of droplets containing different chemical samples that produce precipitates by chemical reaction. Furthermore, to demonstrate droplet manipulation at a high temperature in oil medium, droplets containing a thermochromic ink, which changes color according to the temperature, are transported vertically at different temperatures ranging from 23 °C to 60 °C. Finally, the iodine–starch reaction process is examined as a proof-of-concept of the temperature-controlled chemical reaction on the 3D DMF platform.

## Results

### Droplet detachment on a patterned electrode

In our previous study^35^, in which we utilized a needle-type electrode system, the threshold voltage (or EW number) for droplet detachment in oil was largely reduced by applying a single square signal instead of a DC electrical signal. In the present work, EW actuations with single pulse and DC signals are employed to detach droplets from a patterned electrode submerged in oil medium. Under DC EW actuations, a droplet is detached from a patterned electrode when the applied DC voltage is over 200 V (or *η* > 4.7). To reduce the threshold EW number for droplet detachment, the effect of the applied voltage on the detaching process is investigated by observing the spreading, retracting, and jumping behaviors under EW actuation with a single pulse signal (Fig. 2). The pulse width (T_p_) of the applied square pulse signal is determined based on the results of a preliminary experiment on droplet spreading with varying DC voltages. The spreading time (T_s_, the time to reach the maximum spread radius) of a saline droplet is nearly constant (~12 ms) regardless of the applied DC voltage^27^,^35^. The condition of T_p_ = T_s_ = 12 ms is selected for saline droplets 4 μL in volume to interrupt the electric power supply after the droplet reaches the maximum wetted radius. A droplet is detached from the patterned electrode when *η* is larger than 1.86, which corresponds to the applied voltage of 125 V. That is, the threshold voltage is decreased by about 40% when a droplet is actuated by EW with a single square pulse compared with the corresponding DC EW actuation. This effect may be attributed to the larger amount of surface energy of the droplet electrowetted with a single square pulse voltage compared with that of the corresponding DC voltage. The applied voltage also has little influence on the contact time (i.e., the time elapsed to reach before detaching).

The threshold EW number (*η*_th_) for detaching a droplet in air with DC EW actuations was previously estimated from energy balance between the electrical energy (E_el_) and the adhesion work^27^. Its relation is expressed by *η*_th_ = (1 + cos *θ*_Y_). Here, *θ*_Y_ is the Young’s contact angle of the sessile droplet on a flat solid surface. By applying the experimental conditions of the present study to this relation, the *η*_th_ is estimated to be 0.02, which is about 1% of the experimentally measured value. This discrepancy results from the following reasons. While the *η*_th_ for DC EW actuactions is estimated from the stored surface energy E_s_ at the equilibrium state, that for square pulse EW actuactions should be estimated from the stored energy at the maximum spread state. In addition, this relation does not consider the geometric effects of patterned electrode on the detachment process. For instance, a droplet spreads asymmetrically on the patterned electrode due to its anisotropic geometry, and the contact-line friction and contact-angle hysteresis caused by the step-height of patterned electrode act as the additional dissipation enegy during droplet retraction. Furthermore, it did not consider the inertia effect and viscous dissipation in oil medium. Therefore, a modified relation considering aforementioned factors should be established to accuractely predict the threshold EW number for droplet detachment in oil medium.

### Three-dimensional droplet manipulation

The 3D droplet manipulation is demonstrated by moving a droplet in the vertical and horizontal directions and mixing under EW actuations (Fig. 3). The manipulation consists of five processes: (i) horizontal transport of a sessile droplet, (ii) mixing inside a sessile droplet by oscillation, (iii) upward vertical transport of a sessile droplet, (iv) horizontal transport of a hanging droplet, and (v) downward vertical transport of a hanging droplet. First, to move a 4 μL droplet horizontally on the bottom substrate, a DC voltage with *η* = 0.54 is sequentially applied to the electrodes. Then, an AC voltage with *η*_rms_ = 1.19 at 105 Hz is applied to the electrodes to induce droplet oscillation. Thus, droplets can be rapidly mixed if they contain biochemical samples. Subsequently, the square pulse actuation with *η* = 3.65 and T_p_ = 12 ms induces the droplet to move from the bottom toward the top electrode. A DC voltage with *η* = 0.36 is applied on the top electrode for a short time period (~50 ms) because the droplet detached from the bottom electrode can rebound from the top electrode owing to its hydrophobic wettability. This voltage enables the detached droplet to attach easily to the top electrode. A hanging droplet moves horizontally on the top electrode by adopting the same strategy used for the horizontal transport of a droplet on the bottom substrate. Finally, a square pulse signal with *η* = 3.65 is applied to the electrodes to move the hanging droplet from the top to the bottom electrode. In this step, the buoyance effect is negligible because of the small volume of the droplet and the small difference in densities of the droplet and the ambient oil medium.

### Chemical reaction for precipitate formation

The transport, merging, mixing, and detachment of droplets containing different chemical samples, which produce precipitates by chemical reaction, are examined to demonstrate the applicability of the 3D DMF platform with patterned electrodes to chemical analysis and synthesis (Fig. 4). A yellow powdery precipitate of lead (II) iodide (PbI_2_) is produced when colorless aqueous solutions of lead (II) nitrate [Pb(NO_3_)_2_], and potassium iodide (KI) are mixed together^36^. The chemical equation for precipitate formation reaction is given by


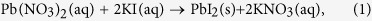


where aq and s in parentheses denote the aqueous solution and the solid in their physical state, respectively. To carry out this chemical reaction on the 3D DMF platform, a 3 μL droplet containing KI (40 mM) is first moved toward another 3 μL droplet containing Pb(NO_3_)_2_ (20 mM). They are merged by sequentially applying a DC electrical signal with *η* = 0.54. The two liquids inside the merged droplet are then mixed rapidly by applying an AC electrical signal with *η*_rms_ = 2.69 at a frequency of 20 Hz. During the mixing of the two liquids, the color of the merged droplet changes sharply from colorless to yellow because of the formation of a yellow powdery precipitate of PbI_2_. After mixing process shown in Fig. 4 (at 3 sec), the two liquids seem to not be well mixed. This is attributed to the fact that the precipitates containing heavy metals such as lead and chromium sink to the bottom of the droplet due to gravity, although the precipitate formation is facilitated by AC EW-driven oscillation. Subsequently, the droplet is vertically moved from the bottom to the top electrode by applying a square pulse signal with *η* = 3.65 and T_p_ = 20 ms. Here, the pulse width T_p_ of the applied square pulse signal is equal to the spreading time T_s_ of the EW-driven spreading droplet 6 μL in volume. Finally, the droplet that hangs from the top electrode moves horizontally and vertically by employing the same strategies used for the horizontal and vertical transports of a droplet on the bottom electrode. Similar to the formation of the PbI_2_ precipitate when aqueous solutions of silver nitrate (AgNO_3_, 100 mM) and potassium chromate (K_2_CrO_4_, 50 mM) are mixed together, a red precipitate of silver chromate (Ag_2_CrO_4_) is produced by the following precipitate formation reaction^36^


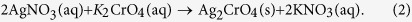


The precipitate formation reaction of Ag_2_CrO_4_ is performed on the 3D DMF platform by employing the same strategies used for the formation of the PbI_2_ precipitate.

### Manipulation of droplets at high temperatures

A main advantage of an immiscible fluid system, such as a water droplet submerged in oil medium, is that the ambient oil prevents droplet evaporation. Thus, the droplet can be manipulated at a high temperature^1^,^32^. To demonstrate the ability of droplet manipulation on the 3D DMF platform at high temperatures, we examine the detachment of 4 μL droplet containing thermochromic ink (4% v/v), which changes color according to temperature at different temperatures that range from 23 °C to 60 °C (Fig. 5a). Even at a high temperature of 60 °C, the 4 μL droplet containing thermochromic ink is moved vertically from the bottom to the top electrode by the square pulse actuation with *η* = 3.65 and T_p_ = 12 ms. The temporal variations of dimensionless volume of the droplets evaporating in ambient air are compared with those in ambient oil to evaluate the effect of ambient oil on the prevention of droplet evaporation (Fig. 5b). Here, the dimensionless volume is defined as the volume of a droplet normalized by its initial volume. During the droplet evaporation for 15 min, the volumes of the droplets in ambient air and ambient oil are reduced, respectively, by 85% and 13% of the initial volume at a high temperature of 60 °C. These results support the finding that a droplet can be manipulated on the 3D DMF platform in an immiscible fluid system at a high temperature with small loss of test samples contained inside a droplet.

### Temperature-controlled chemical reaction

When the iodine solution reacts with starch, it exhibits a purple-black color, the intensity of which decreases with increasing temperature^36^. To carry out this iodine–starch reaction as a temperature-controlled chemical reaction on the 3D DMF platform, we examine the vertical and horizontal transport, merging, mixing, and heating of droplets containing aqueous solutions of starch and iodine (Fig. 6). First, a 3 μL droplet containing starch solution (1 wt%) is moved vertically from the bottom to the top electrode by applying a square pulse signal with *η* = 3.65 and T_p_ = 12 ms (3 s in Fig. 6). Then, the hanging droplet is moved horizontally on the bottom electrode by sequentially applying a DC electrical signal with *η* = 0.54 (5 s in Fig. 6). To merge a 3 μL droplet containing iodine solution (1% v/v) with the 3 μL droplet containing starch solution, the former droplet is moved toward the latter droplet by applying a square pulse signal with *η* = 3.65 and T_p_ = 20 ms (7 s in Fig. 6). Subsequently, the two liquids inside the merged droplet are mixed rapidly by applying an AC electrical signal with *η*_rms_ = 2.69 at a frequency of 80 Hz (9 s in Fig. 6). During the mixing process of the two liquids, the color of the merged droplet changes sharply from colorless to blue-purple. As the temperature of ambient oil increases up to 60 °C, the color becomes faded (39 s in Fig. 6). Based on these results, we will try to synthesize the nanoparticle on the 3D DMF platform and investigate the effect of temperature on the shape and size of nanoparticles in the near future^1^,^37^,^38^,^39^.

## Discussion

To construct a 3D DMF platform with patterned electrodes submerged in oil medium, we first demonstrate the 3D manipulation of a droplet on the platform, vertical and horizontal transport of a droplet, and oscillatory mixing inside a droplet. In the vertical transport of droplets on patterned electrodes, the threshold voltage for the EW actuation with a single square pulse is decreased by about 40% compared to the corresponding DC EW actuation. As a proof-of-concept of nanoparticle synthesis by chemical reaction, the precipitate formations of lead (II) iodide and silver chromate on the 3D DMF platform are examined. In addition, droplets can be moved vertically from the bottom electrode to the top electrode by employing the square pulse actuation even at a high temperature of 60 °C. Compared to a large volume loss of evaporating droplets in ambient air, ambient oil can prevent droplet evaporation; thus, the droplet can be manipulated at high temperatures. Furthermore, the chemical reaction of two droplets containing aqueous solutions of starch and iodine at room and high temperatures is examined to demonstrate the feasibility of temperature-controlled chemical reaction on the 3D DMF platform.

Recently, a 3D cell culture system and a lipid-coated 3D droplet assembly are of great interest in drug discovery, tissue engineering, and stem cell research, because such 3D cellular models can be used to mimic cell-to-cell and cell-to-extracellular matrix interactions, and thus provide more *in vivo*-like microenvironments, compared to conventional 2D cellular models^40^,^41^,^42^. Therefore, 3D cellular models can predict *in vivo* situations in response to external stimuli such as drug dose more accurately. This leads to high-throughput screening for drug candidates with reduced failure rates^43^. In particular, cell spheroids (i.e., multicellular aggregates with spherical shape) are one of the most suitable and well-characterized 3D cellular models for cell study and drug discovery^44^,^45^. To cultivate cell spheroids, various methods, including hanging droplet method, microwell array, and microfluidics, have been developed^42^,^46^,^47^. Among them, the microfluidic-based methods have been recently receiving large attention due to uniform and reproducible spheroid formation, simple handling of liquid such as nutrient supply and medium exchange^47^,^48^,^49^,^50^,^51^. In spite of these technological advantages, their employment to cell-based assays and drug screening process has been still practically limited by difficulties encountered in individual and programmable handling of liquid^47^,^52^. In addition, these methods are not suitable for 3D cell cultures in which precise and selective control of individual spheroids is essentially required^51^,^52^. To alleviate these challenges, the 3D DMF proposed in the present study can be used for 3D cell cultures. To this end, we preliminarily examine the 3D manipulation of 4 μL droplets containing mouse fibroblasts in silicone oil with a viscosity of 0.65 cSt on the 3D DMF platform. The droplet moves horizontally on the bottom electrode by applying a DC voltage of *η* = 2.17 and then moves upwardly by applying square pulses of *η* = 7.46 at T_p_ = 12 ms. Finally, a droplet hanging on the top electrode moves horizontally into the target position by applying the identical signal used for horizontal movement of the bottom substrate, as shown in Fig. 7a. The cells inside the hanging droplet are settled down to the bottom of the droplet within 30 min (Fig. 7b). Thus, a hanging droplet containing cells on the top electrode can be manipulated without adhesion of precipitates to the solid surface and surface contamination. To find optimal experimental conditions for 3D cell cultures using the 3D DMF platform and to evaluate the comparative differences between 2D and 3D DMF platforms in cell cultures, further detailed study will be conducted in the near future.

To further improve the performance of the 3D DMF platform proposed in the present study, we adopt the electro-elasto-capillarity (EEC) which electrically controls the wettability of a droplet on a soft membrane and the encapsulation of a droplet with a soft membrane^53^,^54^,^55^,^56^. It has many technological advantages, such as prevention of evaporation and contamination of liquid. For these reasons, the EEC may have a strong potential in the development of 3D DMF platforms for drug encapsulation and actuation of microelectromechanical devices. To demonstrate applicability of the EEC in a 3D DMF system, we preliminarily examined EEC-driven detachment (or vertical transportation) of droplets wrapped by a flexible thin film both in air and oil mediums (Fig. 8). When square-pulse signals with *η* = 6.18 in air medium and *η* = 12.5 in oil medium are applied between the droplet and the bottom electrode during a constant T_p_ = 7 ms, the flexible film is pulled down towards the substrate during the pulse width of 7 ms. It is then recovered to its original position due to removal of electric field. Consequently, the droplet wrapped by the thin film takes off from the superhydrophobic solid substrate. This results from the conversion of stored elastic energy by Coulomb force and electrowetting into kinetic energy of the droplet. This kinetic phenomenon is similar to jumping of an elastic hoop^57^. A further systematic study about the application of the ECC to the 3D DMF platforms with patterned electrodes is required in the near future.

## Methods

### Sample preparation

Silver nitrate (Sigma-Aldrich), potassium chromate (Sigma-Aldrich), lead (II) nitrate (Daejung Chemicals & Metals Co., Ltd.), and potassium iodide (Daejung Chemicals & Metals Co., Ltd.) are used to carry out reaction experiments on precipitate formation. To examine the feasibility of the temperature-controlled-chemical reaction, iodine–starch reaction with starch powder from potato (Sigma-Aldrich) and povidone-iodine topical solution (Green Phamaceutical Co., Ltd.) is also tested. Thermochromatic ink (Artmgics, Korea) is used to observe the change in color according to temperature variation. The ink color changes according to the temperature as follows: purple in the range of 20 °C to 40 °C, pink in the range of 40 °C to 60 °C, and beige above 60 °C. The temperature of the oil medium is controlled by heating the bottom plate of the 3D DMF platform using a hot plate (PC-420D, Corning). Temperature is measured with an infrared thermometer gun (830-T1, Testo).

### Electrowetting experiment

**Figure 1:**
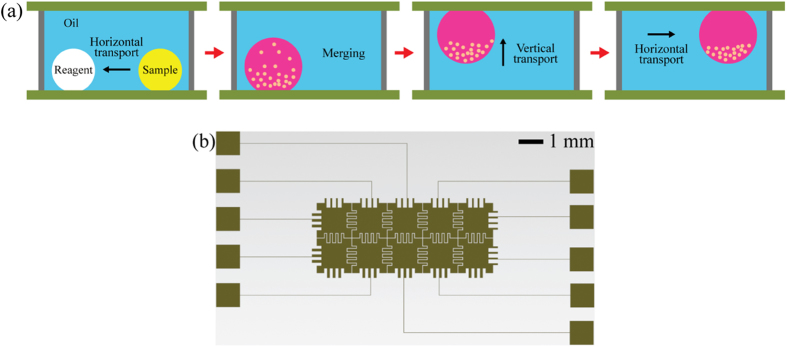
Schematic illustration of the general idea of this study. (**a**) Schematic view of the 3D DMF platform in an oil medium. (**b**) Coplanar interdigitated electrodes as the top and bottom electrodes.

**Figure 2:**
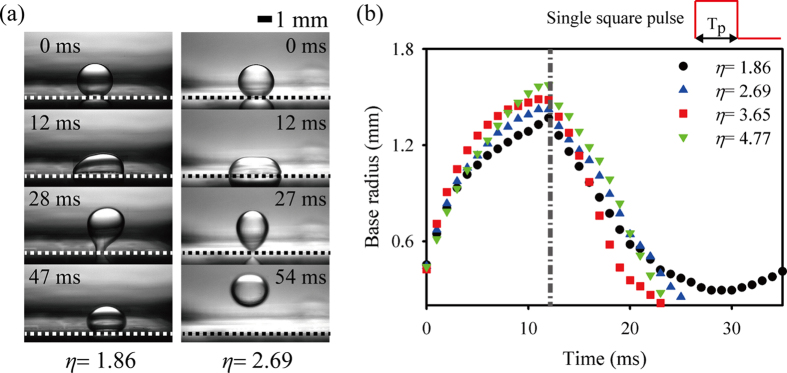
Dynamic behaviors of electrowetted droplets 4 μL in volume in response to square pulse signals with different EW numbers (*η*). (**a**) Consecutive side-view images of droplets under square pulse actuations with *η* = 1.86 and 2.69 at a fixed pulse width (T_p_ = 12 ms). (**b**) Temporal variations of base radius (R_b_) of the droplets electrowetted at different *η*. The dashed and dashed-dotted lines indicate the electrode plate and pulse width, respectively.

**Figure 3:**
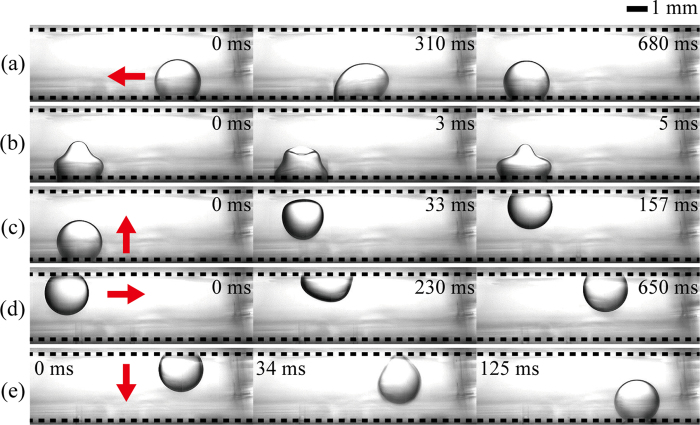
3D manipulation of a droplet by EW actuations. (**a**) Horizontal transport of a sessile droplet, (**b**) mixing inside a sessile droplet by oscillation, (**c**) upward vertical transport of a sessile droplet, (**d**) horizontal transport of a hanging droplet, and (**e**) downward vertical transport of a hanging droplet. The dashed line and arrow indicate the electrode plate and the moving direction of each droplet, respectively.

**Figure 4:**
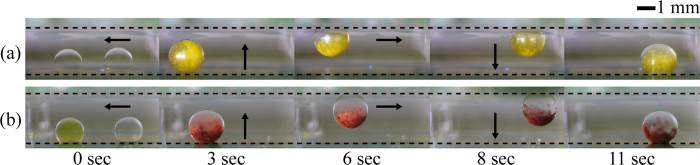
Precipitate formation reaction on the 3D DMF platform with patterned electrodes. (**a**) Formation of a yellow precipitate of lead (II) iodide (PbI_2_). The left and right droplets contain aqueous solutions of lead (II) nitrate [Pb(NO_3_)_2_] and potassium iodide (KI), respectively. (**b**) Formation of a red precipitate of silver chromate (Ag_2_CrO_4_). The left and right droplets contain aqueous solutions of potassium chromate (K_2_CrO_4_), and silver nitrate (AgNO_3_), respectively. The dashed line and arrow indicate the electrode plate and the moving direction of the droplets, respectively.

**Figure 5:**
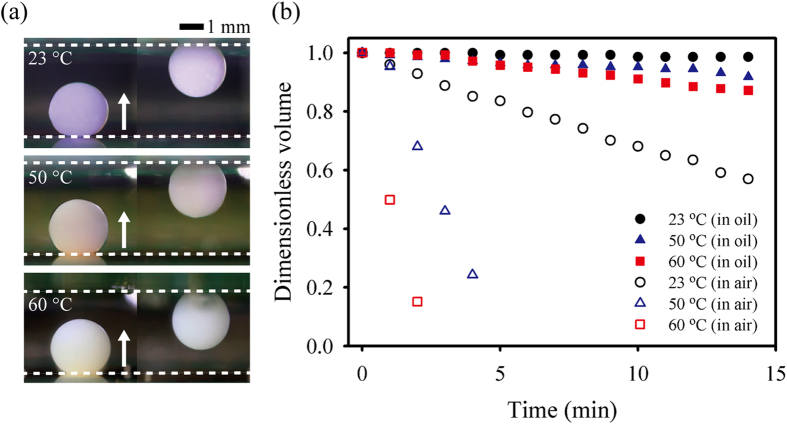
Manipulation of droplets at high temperatures. (**a**) Detachment of droplets containing thermochromic ink in oil medium at temperatures of 23 °C, 50 °C, and 60 °C. The colors of the thermochromic ink are purple, pink, and beige at temperatures of 23 °C, 50 °C, and 60 °C, respectively. (**b**) Temporal variations of dimensionless volume of an evaporating droplet in ambient air (hollow symbol) and ambient oil (filled symbol) at temperatures of 23 °C, 50 °C, and 60 °C. Here, the dimensionless volume is defined as the volume of a droplet normalized by its initial volume. The dashed line and arrow indicate the electrode and the moving direction of each droplet, respectively.

**Figure 6:**
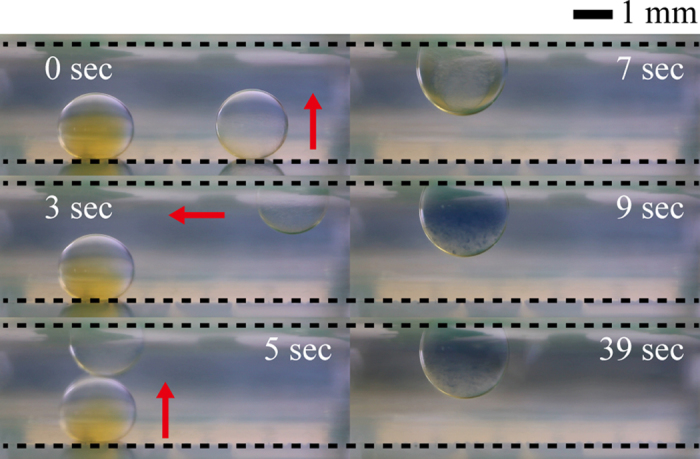
Iodine–starch reaction on the 3D DMF platform. When a droplet containing an iodine solution (left) meets another droplet containing a starch solution (right), the two liquids are mixed rapidly inside a merged droplet, which exhibits a blue-purple color. The intensity of the color decreases with the increase in temperature. The dashed line and arrow indicate the electrode plate and the moving direction of each droplet, respectively.

**Figure 7:**
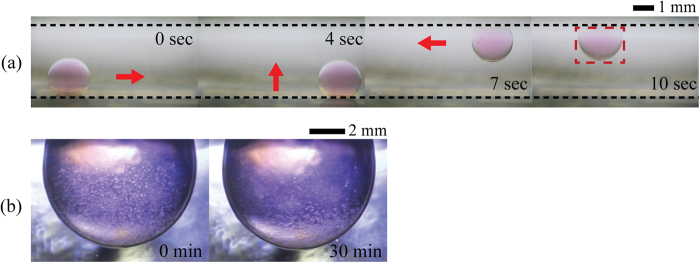
3D manipulation of a droplet containing bio-cells. (**a**) 3D manipulation of 4 μL droplets containing mouse fibroblasts in three steps: (i) horizontal transport on the bottom substrate by applying a DC voltage of *η* = 2.17, (ii) vertical transport by applying square pulses of *η* = 7.46 at T_p_ = 12 ms, and (iii) horizontal transport on the top substrate by applying a DC voltage of *η* = 2.17. Red arrows and black dashed lines indicate the moving direction of each droplet and the electrode plates, respectively. (**b**) Close-up of the bottom part of hanging droplet containing cells. The cells inside the hanging droplet are settled down to the bottom of the droplet within 30 min.

**Figure 8:**
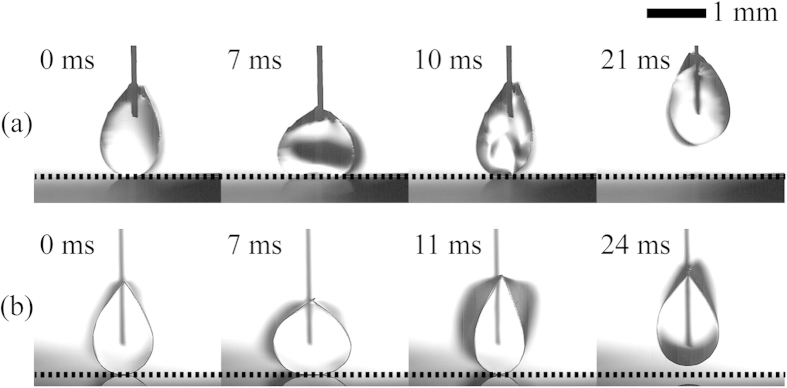
EEC-driven detachment (or vertical transportation) of droplets wrapped by a flexible thin film both in air and oil medium. Temporal shape variations of detaching 7 μL droplets wrapped by a flexible parylene-C film (5 μm thickness, 5 × 2 mm rectangular slices) in (**a**) air by applying square-pulse signal with *η* = 6.18 and T_p_ = 7 ms, and in (**b**) silicone oil with a viscosity of 0.65 cSt by applying square-pulse signal with *η* = 12.5 and T_p_ = 7 ms.

The experimental apparatus mainly consists of two parallel plates attached to the same patterned electrode array (Fig. 1). The patterned electrode array is fabricated as follows. A glass wafer (Pyrex® 7740, Corning) is coated with a 20 nm-thick layer of chromium as an adhesion layer and a 200 ± 10 nm-thick layer of gold as an electrode through electron beam deposition. A photoresist (AZ 5214, AZ Electronic Materials) is patterned on the gold by photolithography. The gold and chromium are patterned in sequence by wet etching. After the remaining photoresist is removed, a 5 ± 0.5 μm-thick insulating layer of parylene-C is deposited by vapor deposition using a parylene coating system. The patterned electrode array is composed of 2 × 5 electrodes (Fig. 1b inset), and the gap between two adjacent electrodes is 20 μm in width. The physical dimension of one digitized patterned electrode is 1.5 mm × 1.5 mm. On the top of the insulation layer, Teflon AF1600® (DuPont) is spin-coated in 100 nm thickness to make the surface hydrophobic. The bottom electrode plate is embedded in an acrylic cell 3 cm long, 2.5 cm wide, and 3 cm high. The cell is filled with silicone oil (Shin-Etsu Silicone Korea Co., Ltd.), which has a kinematic viscosity of 0.65 cSt. An aqueous 0.1 M NaCl solution is used as the conducting liquid. Interface tension between saline solution and silicone oil is measured as 0.020 N/m^58^. The data provided by the supplier show that the density of the tested silicone oils with a viscosity of 0.65 cSt is 0.76 g/mL. A droplet of 3 μL or 4 μL saline solution is dispensed with a micropipette onto the bottom electrode. Then, all the devices are assembled with the top and bottom electrodes separated by spacers made of acryl column 3.0 ± 0.1 mm in height.

DC electrical signals are generated by a function generator (33220A, Agilent) and then amplified (PZD700, Trek). The applied voltage signals (*V*_a_) are ranged from 100 V to 400 V for both the horizontal and vertical transports. In this study, the dimensionless EW number *η* = *ε*_d_*ε*_o_*V*^2^/2*dγ* is used as one of the governing parameters instead of the applied voltage. This number represents the relative strength of electrostatic energy compared to the surface tension^7^. Here, *ε*_d_ and *d* are the dielectric constant and insulator thickness, respectively; *ε*_o_ is the vacuum dielectric permittivity, *V* is the applied voltage, and *γ* is the interfacial tension between liquids. Note that *V* is the voltage between the liquid at the contact line and the adjacent electrode, so in our experimental setup, *V* = *V*_a_/2. The values of *ε*_d_ = 3.1, *ε*_o_ = 8.854 × 10^−12^ F/m, and *d* = 5 μm are used to evaluate the EW number. Electrical signals are transmitted to the electrodes through photo-coupled relays (AQW216, Panasonic) controlled by a digital I/O board (Mega 2560, Arduino) along with a programmed LabVIEW^®^ code.

Dynamic behaviors of the electrowetted droplets are consecutively recorded with a high-speed camera (Fastcam SA3, Photron) or a digital camera (Canon EOS 500D). Digital image processing and data analysis are performed using MATLAB® and a public domain image-processing program (ImageJ, NIH). Each experiment under the same experimental condition is repeated five times, and all the results are presented as the statistical average.

### Cell preparation

For the 3D manipulation of droplets containing cells, NIH/3T3 mouse embryonic fibroblasts are cultured in Dulbecco’s modified eagle medium (DMEM) (Gibco), supplemented with 10% BCS (Gibco) and 0.1 mg/mL penicillin-streptomycin (Gibco) under a humidified atmosphere of 5% CO_2_/95% air at 37 °C. The cultured fibroblasts are washed with Dulbecco’s phosphate buffered saline and detached from the flask with trypsin–EDTA solution. The detached cells are then pelleted by slow centrifugation and resuspended in DMEM. Consequently, the sample of fibroblasts is diluted to a final concentration of 5 × 10^4^ cells/mL.

### Detachment of a droplet by means of electro-elasto-capillarity

The experimental setup employed in the present study is a typical apparatus used for the EW experiment with the needle electrode configuration. A flexible parylene-C film (5 μm thickness, 5 × 2 mm rectangular slices) is placed on a superhydrophobic surface. The superhydrophobic substrate was fabricated as following procedure: (1) employing metal-assisted chemical etching (MACE) of Si wafer in HF/H_2_O_2_/AgNO_3_ system to fabricate nanostructures at room temperature, and (2) coating of a self-assembled monolayer (SAM) with low surface energy. More detailed information is available in reference 59. When a 7 μL droplet of saline solution (0.1 M NaCl solution) is dispensed onto the flexible parylene-C film, the thin film starts to bent upward and then completely wraps around the droplet due to the vertical component of the liquid–vapor interface tension. A tungsten wire with a diameter of 80 μm is immersed in the test droplet as a top electrode. Electrical signals produced by a function generator (33220A, Agilent) and amplifier (PZD700, Trek) are applied between a tungsten wire and an electrode plate beneath the superhydrophobic surface. The experimental setup and procedure for detachment of a droplet in oil medium are almost the same as those in air medium. Silicone oil with a kinematic viscosity of 0.65 cSt is used as surrounding oil phase. Without applied voltage, a droplet on a flexible parylene-C film in oil medium has a contact angle around 150 degrees, which is larger than one in air medium. Thus, the droplet is hardly encapsulated by the parylene-C film. To solve this problem, a parylene-C film treated by oxygen plasma at 40 W for 90 sec is used for the case of oil medium.

## Additional Information

**How to cite this article**: Hong, J. *et al*. Three-dimensional digital microfluidic manipulation of droplets in oil medium. *Sci. Rep.* **5**, 10685; doi: 10.1038/srep10685 (2015).
